# LPI-GPR55 promotes endothelial cell activation and inhibits autophagy through inducing LINC01235 expression

**DOI:** 10.1080/07853890.2024.2407525

**Published:** 2024-09-24

**Authors:** Xiaoying He, Xin Zhao, Hongqin Wang

**Affiliations:** a Shanxi Provincial Key Laboratory of Kidney Disease, Shanxi Provincial People’s Hospital, Taiyuan, China; b Yaodu District People’s Hospital, Linfen, China

**Keywords:** Lysophosphatidylinositol, LINC01235, miRNA-224-3p, RABEP1, autophagy

## Abstract

**Introduction:**

Atherosclerosis (AS) is a chronic inflammatory disease characterized by lipid accumulation, inflammation and apoptosis of the arterial wall. This study evaluated the effects of lysophosphatidylinositol (LPI) on endothelial cells activation and autophagy in AS.

**Methods:**

qRT-PCR and Western blotting were done to verify the expression of ICAM1, GPR55 and SOD2. RNA-Seq was performed and screened for the different expressions of long noncoding RNAs (lncRNAs), combining bioinformatics analysis to elucidate the mechanism by which lncRNA functions.

**Results:**

qRT-PCR and Western blotting results showed that LPI increased GPR55 and ICAM1 expression. RNA-Seq analysis and qRT-PCR results showed that LPI increased the expression of LINC01235, LINC00520 and LINC01963; LINC01235 was the most obvious. Mechanistically, bioinformatic analysis demonstrated that LINC01235 inhibited autophagy through sponging miR-224-3p. And miRNA-224-3p targeted RABEP1.

**Conclusions:**

LPI promoted endothelial cell activation. LPI induced the expression of LINC01235 and LINC01235 inhibited autophagy through miR-224-3p/RABEP1. Collectively, this study first reveals the function of LINC01235, which may serve as a potential therapeutic target in AS.

## Introduction

Atherosclerosis (AS) is a chronic inflammatory disease characterized by lipid accumulation, inflammation and apoptosis of the arterial wall. Atherosclerosis begins with the development of vascular endothelial dysfunction. Many factors (such as oxidative stress and hyperlipidaemia, etc.) can cause endothelial cell dysfunction. As a new generation of lipids, lysophospholipids (LPLs), such as lysophosphatidic acid (LPA), lysophosphatidylserine (LysoPS) and lysophosphatidylinositol (LPI), have gradually attracted the attention of many researchers. Studies have proved that abnormal lipid metabolism increases endogenous LPLs, and the increased LPLs, as the Danger-associated molecular patterns (DAMPs), promote the activation of endothelial cells, induce inflammation, and ultimately promote the occurrence of AS [1]. LPI is a metabolic intermediate of phospholipids. LPI is a biological lipid produced by PLA [1]. LPI is an endogenous ligand for GPR55 [2]. Studies have shown that LPI plays a role in non-alcoholic fatty liver, ischemia/reperfusion (I/R) injury and obesity [3,4]. Although research has shown that LPI induces a rapid and transient increase in [Ca^2+^]*i* in cardiomyocytes through an action at GPR55 receptors located on both the sarcolemma and the membranes of intracellular organelles [2], its role in endothelial cells is unknown. At present, the research on LPI mostly focuses on the LPI-GPR55 signalling pathway, and whether other factors are involved in LPI-induced AS remains unclear.

The length of long noncoding RNAs (lncRNAs) is greater than 200 nt. Studies have shown that lncRNAs can participate in various pathological and physiological processes [5]. In particular, lncRNA plays an important role in cardiovascular diseases. Some lncRNAs are associated with endothelial dysfunction and vascular inflammation, such as lncRNA TIE-1AS, FA2H-2, MALAT1, SENCR and VINAS [6–10]. They regulate target gene expression at the transcriptional and post-transcriptional levels. LncRNAs combined with miRNA to degrade target genes. In our study, we found that LPI increased the expression of LINC01235, LINC00520 and LINC01963, but the underlying mechanism needs to be clarified.

RABEP1 (Rab GTPase-binding effector protein 1) is an extended coiled-coil protein. RABEP1 has three binding sites: RAB4, RAB5 and clathrin coat adaptors AP-1 and GGA [11]. Studies have shown that RABEP1 forms a complex with RB1CC1/FIP200 and ATG16L1. RABEP1 induces selective autophagy in endosome [9]. RABEP1 is involved in KCNH1 channels trafficking to and from the cell membrane [9]. To date, research on RABEP1 has mainly focused on endosomes. In our study, we uncovered a new role for RABEP1 in the regulation of endothelial function.

## Materials and methods

### Cell culture

In this study, we used human aortic endothelial cells (HAECs: ATCC^®^ PCS-100-011, accession no. CVCL_C0EQ) as VEC model. HAECs were grown in vascular cell basal medium (ATCC^®^ PCS-100-030) with an endothelial cell growth kit-VEGF (ATCC^®^ PCS-100-041) in a humidified incubator at 37 °C with 5% CO_2_. HAECs in our study have been tested and found free of mycoplasma.

### siRNAs transfection

Duplex oligonucleotides were chemically synthesized and purified by GenePharma (Shanghai, China). The small interfering RNA (siRNA) duplexes used were LINC01235, sense: 5′-GGGAGAGAAACCCGAAUAUTT-3′, antisense: 5′-AUAUUCGGGUUUCUCUCCCTT-3′, cells were transfected with siRNA duplexes using Lipofectamine 2000 (11668-019, Invitrogen, Waltham, MA) according to the manufacturer’s instructions.

### Western blotting

After treatment, HAECs were lysed in RIPA lysis buffer (P0013B, Beyotime, Shanghai, China). Protein content was determined using a BCA Protein Assay Kit (P0011, Beyotime, Shanghai, China). Proteins were separated by 12% SDS-PAGE at 4 °C and then transferred to PVDF membrane (IPFL00010, Millipore, Burlington, MA). The membrane was incubated with primary antibodies: GPR55 (9542S, Cell Signaling Technology, Danvers, MA); ICAM1 (9662S, Cell Signaling Technology, Danvers, MA); LC3B (9272, Cell Signaling Technology, Danvers, MA); and β-actin (A-5441, Sigma, St. Louis, MO) at 4 °C overnight and detected with the corresponding horseradish peroxidase-conjugated secondary antibody (1:10,000) at room temperature for 1 h. The membranes were incubated with Immobilon Western Chemiluminescent HRP Substrate for 5 min and then exposed with Gel Image Station (ChemiDoc, Bio-Rad, Melville, NY). The relative protein content was analysed using Image J software (Bethesda, MD) and normalized to the loading controls.

### Quantitative real-time PCR

The total RNAs were extracted from HAECs using Trizol reagent method (TAKARA, 9109, Kyoto, Japan). According to the PrimeScript RT reagent kit’s (TAKARA, DRR047, Kyoto, Japan) protocol, the RNA was reserved to cDNA. Quantitative RT-PCR reactions involved the use of SYBR Premix Ex Taq (Tli RNaseH Plus) and were carried out in a 20 μL volume with 10 μL of 2X SYBR Green I, 0.4 μM sense primer, 0.4 μM antisense primer, 1 μg cDNA template and 7.2 μL distilled water. Relative gene expression was normalized to U6.

### Cell adhesion analysis

HAECs were seeded in a six-well plate and then treated with LPI for 18 h. Simultaneously, THP-1 cells were incubated with 2 μM Calcein AM for 30 min, marking them as Calcein AM-positive cells. Then, the labelled THP-1 cells were added to the treated HAECs and cultured at 37 °C for 1 h. Cells were washed with culture medium, and THP-1 cells that were not bound to HAEC were discarded. Using the fluorescence microscope to measure the changes in fluorescence, we calculated the ratio of THP-1 cells adhering to HAECs.

### Immunofluorescence detection of mtROS

HAECs were treated with LPI for 18 h and incubated with 5 μM MitoSOX Red for 20 min, using the fluorescence microscope to observe changes in fluorescence. The experiment was repeated at least three times and selected at least three different fields for each group.

### Statistical analysis

Data are presented as mean ± SD, and analysis involved use of GraphPad Prism 7 (GraphPad Software, La Jolla, CA). Differences between two groups were compared using Student’s *t*-tests. Normal distributions were assessed using the Shapiro–Wilk test. Differences among multiple groups were compared using one-way ANOVA followed by Tukey’s post hoc analysis. Images were processed using Adobe Photoshop CC software (Adobe, San Jose, CA). *p* < .05 was considered statistically significant. All experiments were independently repeated at least three times.

## Results

### LPI targeted GPR55 and promoted endothelial cell activation

**Figure 1.**
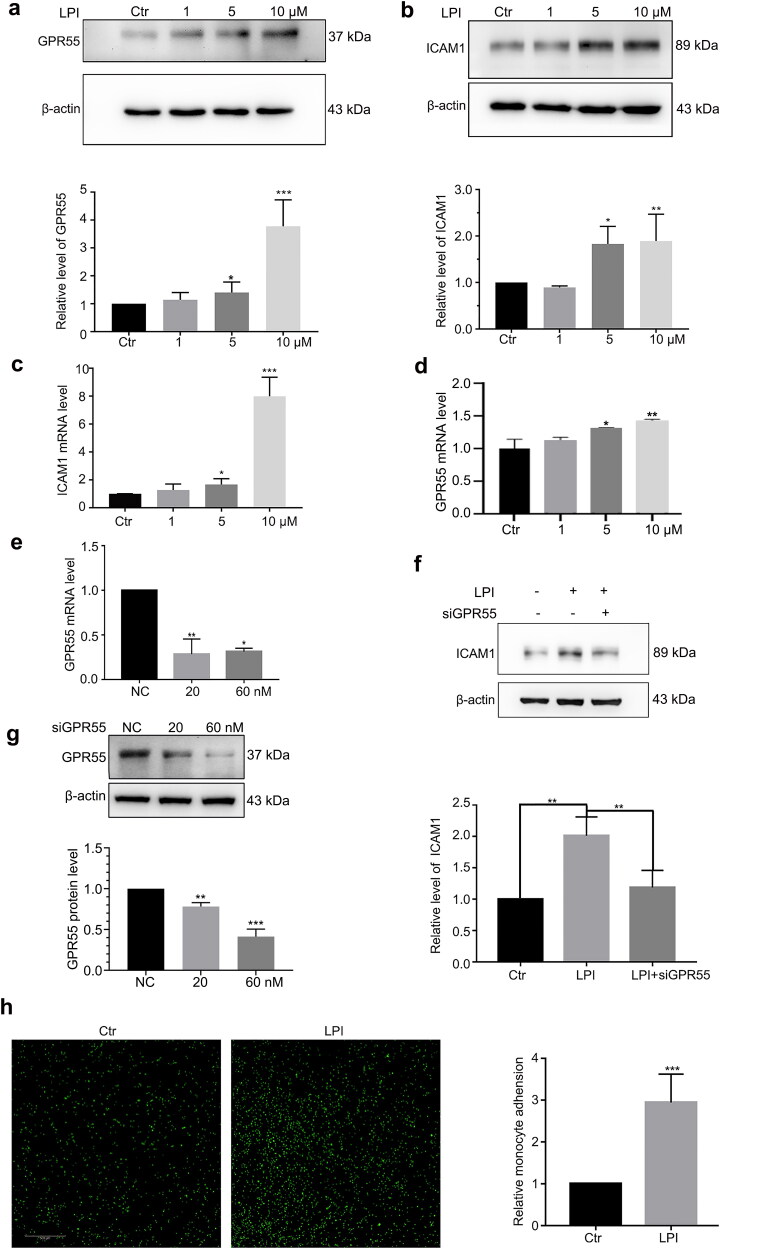
LPI targeted GPR55 and promoted endothelial cell activation. (a, b) Western blot analysis of GPR55 and ICAM1 protein levels after treating with LPI (1, 5 and 10 μM) for 18 h. (c, d) RT-PCR analysis of ICAM1 and GPR55 mRNA levels after treating with LPI (1, 5 and 10 μM) for 18 h. (e, g) HAECs were transfected with siGPR55 at 20 and 60 nM for 24 h. RT-PCR and Western blot analysed the RNA and protein level of GPR55. (f) HAECs were transfected with siGPR55 (60 nM) for 24 h and then treated with LPI (10 μM) for 18 h. Western blot analysed ICAM1 protein level. (h) Immunofluorescence analysed the adhesion of monocytes to endothelial cells after treating with LPI (10 μM) for 18 h, scar bar: 500 μm. Data are represented as mean ± SD (*p < .05, **p < .01 and ***p < .001, *n* = 3).

**Figure 2.**
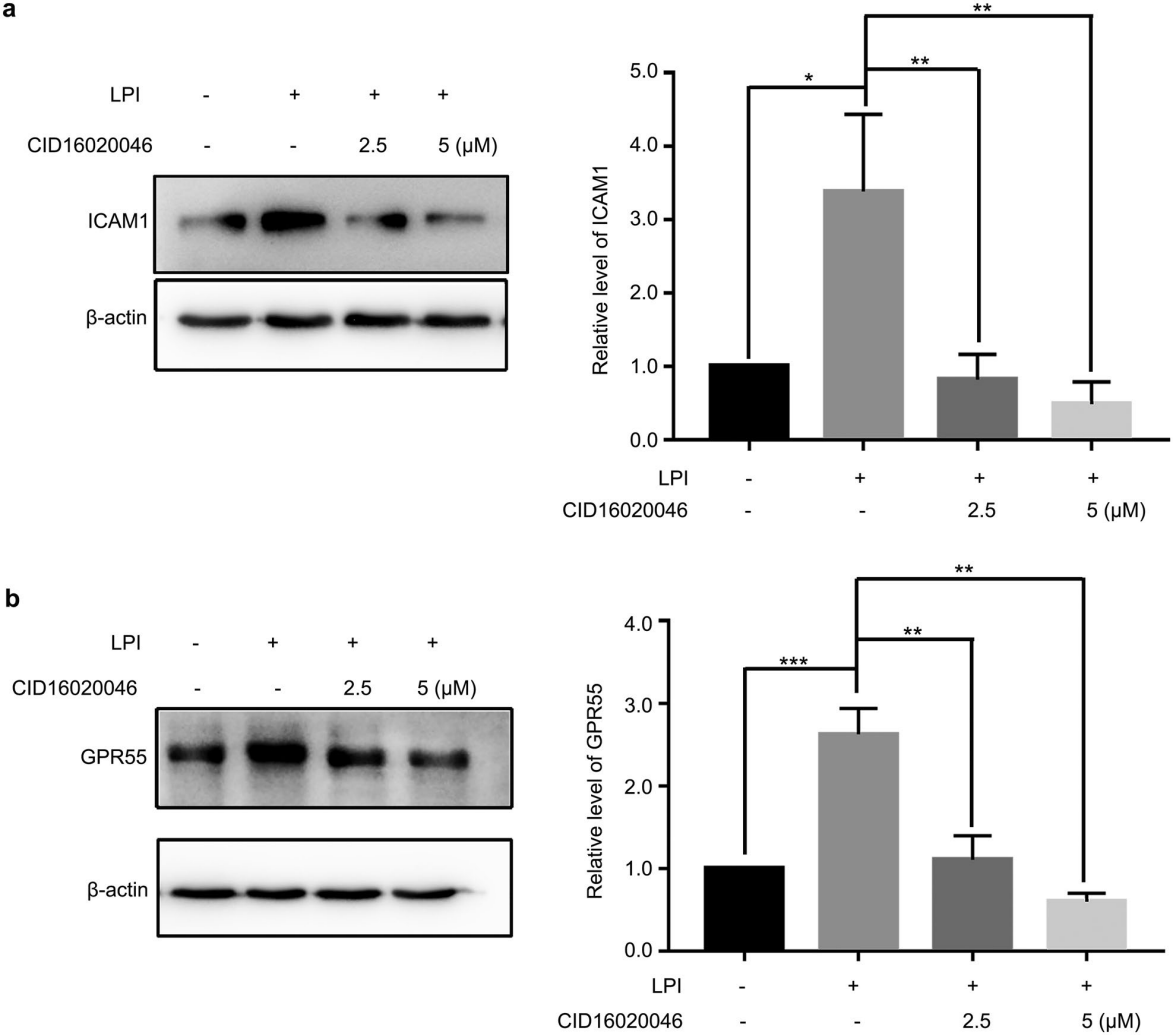
Blocking the function of GPR55, LPI no longer increased ICAM1. (a, b) HAECs were treated with different concentrations of CID16020046 (2.5 and 5 μM) for 24 h and then treated with LPI (10 μM) for 18 h. Western blot analysed ICAM1 and GPR55 protein levels. Data are represented as mean ± SD (*p < .05, **p < .01 and ***p < .001, *n* = 3).

To detect the role of LPI in endothelial cell activation, we treated endothelial cells with different concentrations of LPI. ICAM1 (intercellular adhesion molecule 1) is a marker of endothelial cell activation. Western blot and RT-PCR analysis showed that LPI increased the protein and RNA level of GPR55 (Figure 1(a,d)). Meanwhile the protein and RNA levels of ICAM1 also increased (Figure 1(b,c)). The adhesion of monocytes to endothelial cells experiment demonstrated that LPI promoted this effect (Figure 1(h)). GPR55 is the only endogenous receptor of LPI. Therefore, we synthesized specific siRNAs against GPR55, and HAECs were transfected with si*GPR55* at 20 and 60 nM. The efficiency of RNAi was checked by Western blotting and qPCR (Figure 1(e,g)). We blocked the function of GPR55 using its siRNA; at this time, LPI no longer increased ICAM1 expression (Figure 1(f)). CID 16020046 is an effective, selective GPR55 antagonist. Using CID 16020046 to block the action of GPR55, the same results were obtained by Western blotting (Figure 2(a,b)). Above all, LPI promoted endothelial cell activation.

### LPI promoted mtROS through decreasing the activity of SOD2

**Figure 3.**
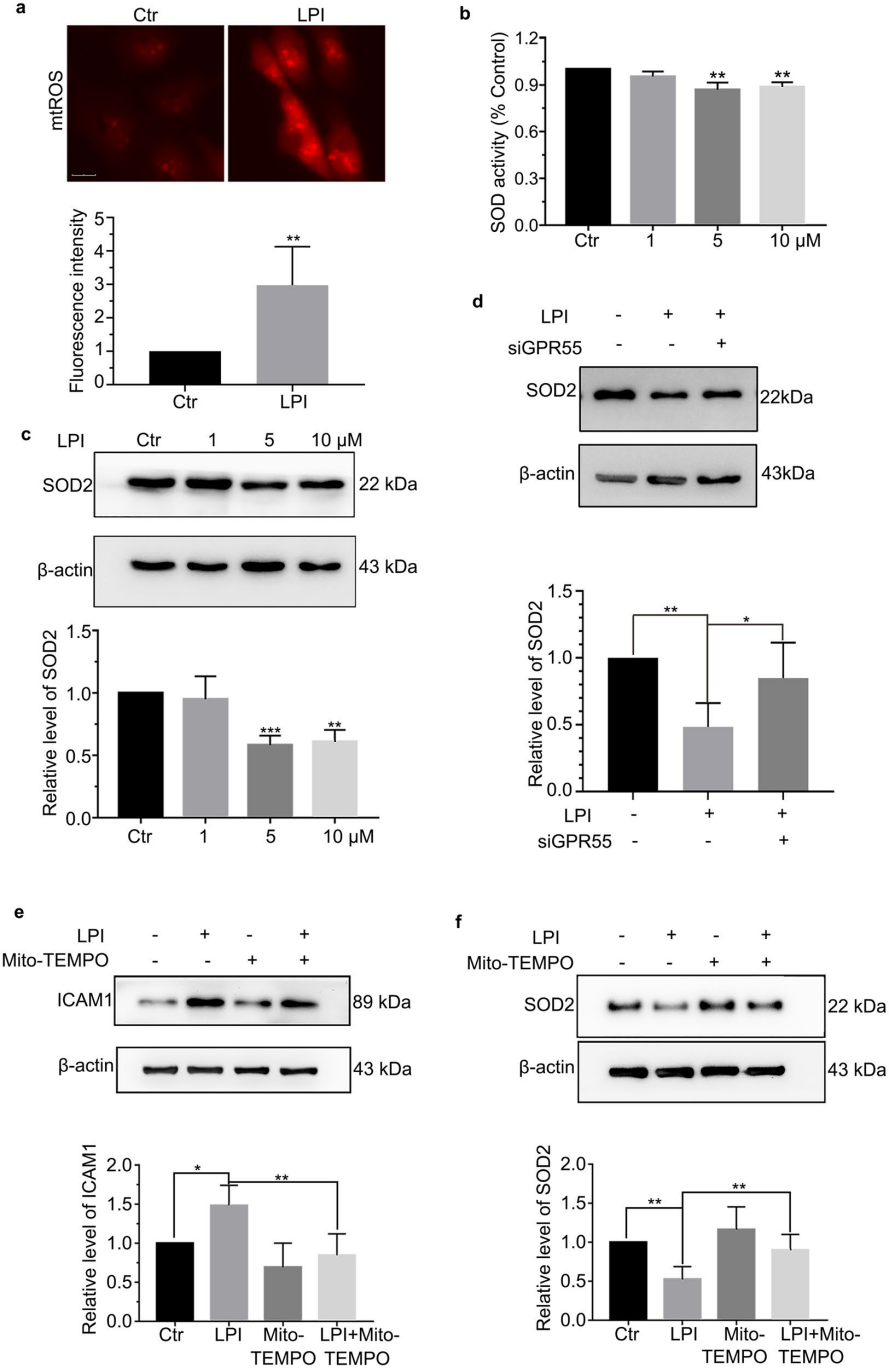
LPI promoted mtROS through reducing the activity and protein level of SOD2. (a) Immunofluorescence detected mtROS level after treating with LPI (10 μM) for 18 h, scale bar: 25 μm. (b) Using the reagent kit to detect SOD activity after treating with LPI (1, 5 and 10 μM) for 18 h. (c) Western blot analysis of SOD2 protein level after treating with LPI (1, 5 and 10 μM) for 18 h. (d) HAECs were transfected with siGPR55 (60 nM) for 24 h and then treated with LPI (10 μM) for 18 h. Western blot analysed SOD2 protein level. (e, f) HAECs were pretreated with mito-TEMPO (10 μM) for 2 h and then treated with LPI (10 μM) for 18 h. Western blot analysed ICAM1 and SOD2 protein level. Data are represented as mean ± SD (*p < .05, **p < .01 and ***p < .001, *n* = 3).

Next, we explored the reasons for the endothelial cell activation. mtROS is the main component of intracellular ROS. Previous studies have shown that endothelial cell mtROS can activate endothelial cells, leading to the recruitment of inflammatory cells in physiological or pathological states. Therefore, we detected whether LPI induced mtROS. Immunofluorescence results indicated that LPI promoted mtROS (Figure 3(a)). As a superoxide dismutase, SOD2 plays an important role in the antioxidant processes. Once there is an imbalance between oxidation and antioxidation, the cells produce excess ROS. Therefore, we detected the protein level of SOD2 and its activity. The results showed that LPI decreased SOD2 protein level and activity (Figure 3(b,c)). Mito-TEMPO is a mitochondria-targeted superoxide dismutase mimetic with superoxide and alkyl radical scavenging properties [12]. We blocked the production of mtROS using mito-TEMPO; LPI no longer decreased SOD2 and the expression of ICAM1 no longer increased (Figure 3(e,f)). At the same time, the same result was obtained after knocking down GPR55 (Figure 3(d)). Collectively, our results showed that LPI promoted endothelial cell activation mainly by inducing mtROS.

### LPI promoted LINC01235 expression

**Figure 4.**
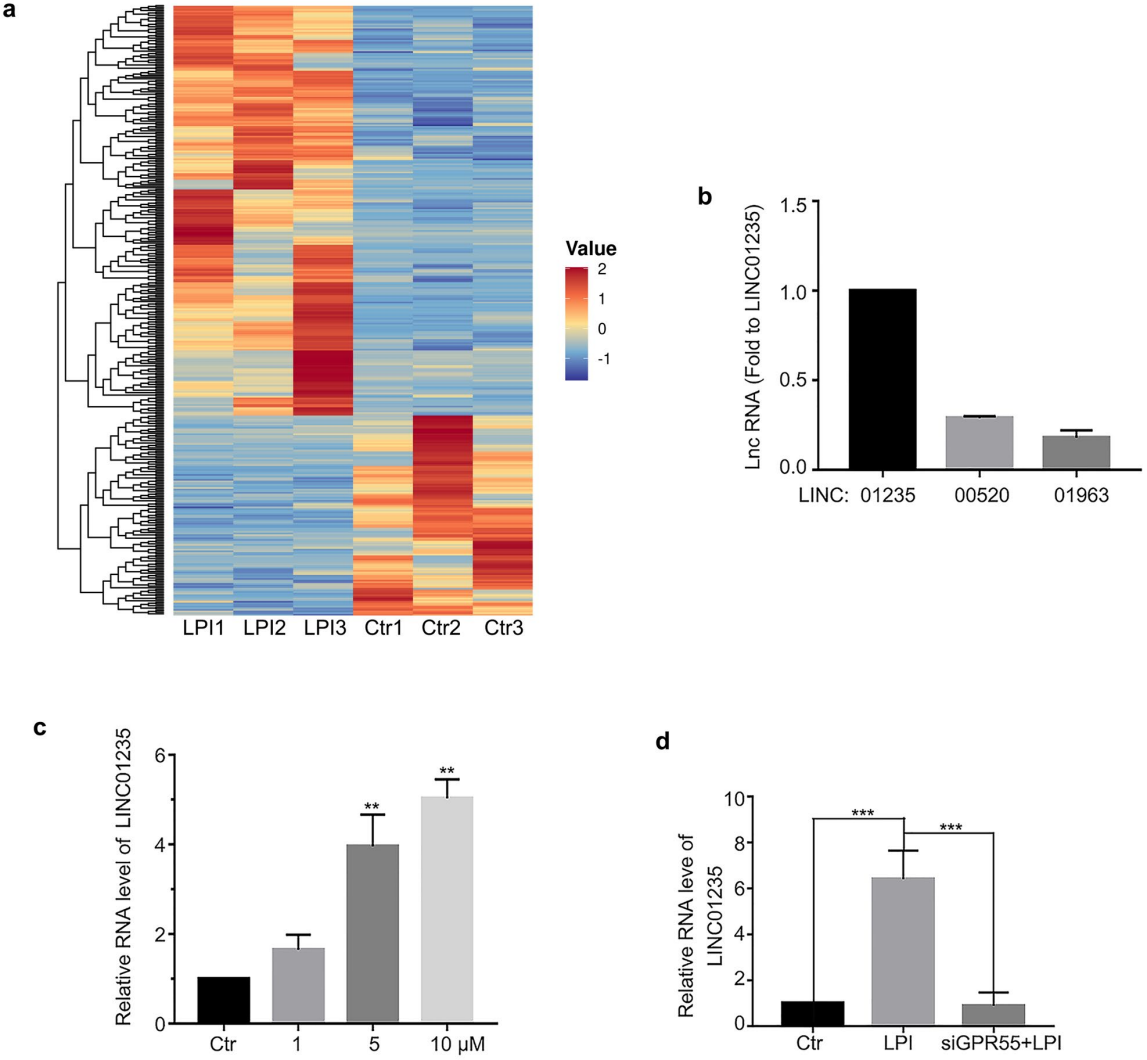
LPI promoted the expression of LINC01235. (a) Gene cluster analysis was conducted based on the FPKM value of each sample. The *X*-axis represents the different samples, whereas the *Y*-axis represents DEGs. The colour (from blue to red) represents DEG expression intensities from low to high. (b) Based on these results, we verified the significantly upregulated genes. (c) RT-PCR analysed LINC01235 expression after LPI treatment for 18 h. (d) HAECs were transfected with siGPR55 (60 nM) for 24 h and then treated with LPI (10 μM) for 18 h. RT-PCR analysed LINC01235 expression. Data are represented as mean ± SD (**p < .01 and ***p < .001, *n* = 3).

To determine the mechanism of LPI, we conducted RNA-Seq analysis. The results showed that LPI increased the expression of the three lincRNAs (Figure 4(a)). Using qPCR, we demonstrated that LINC01235, LINC00520 and LINC01963 expression increased after LPI treatment (Figure 4(b)), and LINC01235 was the most obvious. Meanwhile, LPI promoted LINC01235 expression in a concentration-dependent manner (Figure 4(c)). Knocking down GPR55, LINC01235 no longer increased (Figure 4(d)). Taken together, our data demonstrated that LPI promoted the expression of LINC01235.

### LINC01235 suppressed HAECs autophagy via sponging miR-224-3p

**Figure 5.**
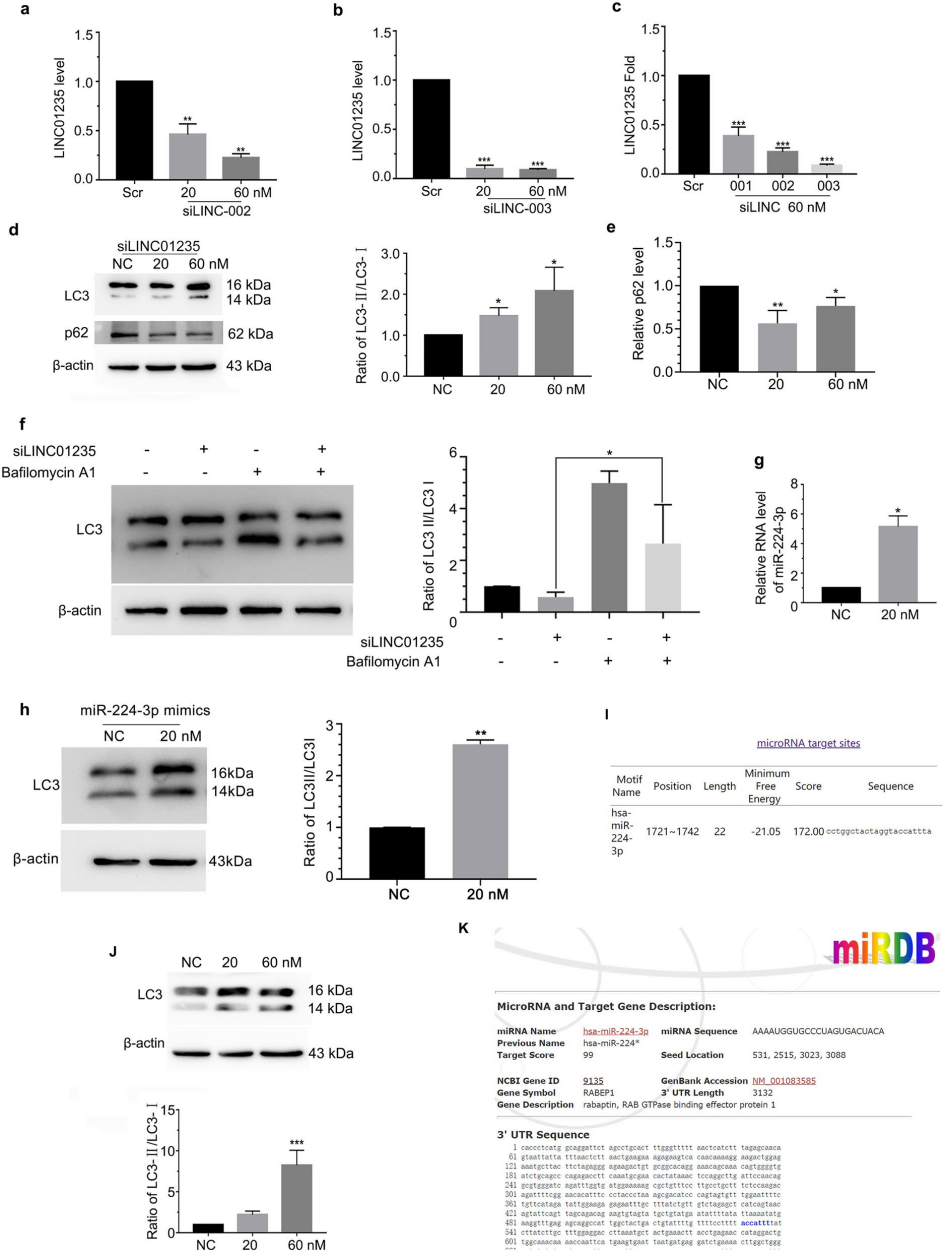
LINC01235 sponging miR-224-3p and then suppressed autophagy. (a–c) Designed and synthesized three siRNAs (001, 002 and 003), HAECs were transfected with siLINC (20 and 60 nM) respectively for 24 h and then RT-PCR analysed LINC01235 expression. (d, e) HAECs were transfected with siLINC01235 (20 and 60 nM) for 24 h and LC3 and p62 protein level were analysed by Western blotting. (f) HAECs were transfected with siLINC01235 for 48 h, followed by treatment with BafA1. Western blot analysed LC3 protein level. (g) HAECs were transfected with siLINC01235 for 24 h and then RT-PCR analysed miR-224-3p expression. (h) HAECs were transfected with miR-224-3p mimics for 48 h. Western blot analysed LC3 protein level. (i) Bioinformatics analysis of LINC01235 and miR-224-3p binding sites. (j) HAECs were transfected with siRABEP1 (20 and 60 nM) for 48 h and LC3 protein levels were analysed by Western blotting. (k) Bioinformatics analysis of miR-224-3p binding sites. Data are represented as mean ± SD (*p < .05, **p < .01 and ***p < .001, *n* = 3).

Autophagy plays an important role in endothelial cell activation. Therefore, we investigated the role of LINC01235 in autophagy in the present study. We synthesized specific siRNAs against LINC01235, and HAECs were transfected with siLINC01235 at 20 and 60 nM. The efficiency of RNAi was verified using qPCR (Figure 5(a–c)). When siLINC01235 blocked the function of LINC01235, the level of autophagy was elevated, as indicated by the upregulation of LC3 and the downregulation of p62 (Figure 5(d,e)). To further verify the integrity of autophagy flow, bafilomycin A1 was used to block the fusion of autophagosomes and lysosomes. Results showed that LC3 protein level still increased after blocking the autophagic flux (Figure 5(f)). Studies have shown that lncRNAs affect mRNA stability by competing with endogenous RNAs (ceRNAs) [13]. To determine the mechanism by which LINC01235 inhibits autophagy in HAECs, we predicted miRNAs that may combine with LINC01235 using bioinformatics analysis (RegRNA2.0: http://regrna2.mbc.nctu.edu.tw/detection.html) and found that miR-224-3p had higher binding scores (Figure 5(i)). LINC01235 knockdown elevated the level of miR-224-3p (Figure 5(g)). Furthermore, we predicted the possible sites for miR-224-3p bonding to LINC01235, the putative sites at positions 1721-1742 (Figure 5(i)).

### miR-224-3p targeted the 3′ UTRs of RABEP1 and RABEP1 suppressed autophagy

Studies had shown that miR-224-3p targeted MTDH and then contributed to CDDP resistance of oesophageal squamous cell carcinoma. miRNA-224-3p also targets JAK1 during AS [14]. In our study, we found that miRNA-224-3p sponges RABEP1 and LC3 protein level was elevated after transfecting with miR-224-3p mimics (Figure 5(h,k)). RABEP1 participants in early endosome homeostasis [11], but there is no study of RABEP1 in AS. Therefore, we investigated the role of RABEP1 in autophagy. We used siRABEP1 to block the function of RABEP1, and found that the level of autophagy was elevated (Figure 5(j)). Above all, our data demonstrated that miRNA-224-3p promotes autophagy in HAECs by targeting RABEP1.

## Discussion

Endothelial cells are the first barrier of blood vessels. Thus, endothelial cell function is very important in AS. LPI is the product of lipid metabolism. As the DAMPs, LPI induces inflammation and contributes to AS. LPI functions as an endogenous agonist of GPR55. Studies have showed that GPR55 was expressed in osteoclasts, osteoblasts, macrophages, monocytes and B-cells [15,16]. GPR55 plays different roles in different cells. In splenic B cells, GPR55 is highly expressed and has an impact at early stages of B cell differentiation. GPR55 signalling exerts an atheroprotective effect in B cells [15]. However, in AS initiation and progression, GPR55 is highly expressed in human inflammatory cells [16]. GPR55 also promotes AS in endothelial cells and macrophages [17,18], which is consistent with our research. LPI-GPR55 signal pathway was involved in various physiological processes. LPI induces cell rounding and stress fibre formation in a GPR55-dependent manner [19]. LPI activates murine and human‐induced pluripotent stem cell cardiomyocytes via a GPR55/RhoA/ROCK/p38 MAPK‐dependent pathway [2]. In AS, CID16020046 blockage of GPR55 could suppress monocyte adhesion through suppression of Mac-1 expression [20]. In our study, we found that LPI promoted HAEC activation by increasing the expression of ICAM1. Activated endothelial cells promote the development of AS.

Growing evidence has demonstrated that ROS plays a vital role in the regulation of endothelial cell function. Previous studies have shown that LPC activates mtROS in endothelial cells [21]. However, there have been no reports of LPI-mediated endothelial cell activation. In our study, we used fluorescence microscopy to detect intracellular mtROS levels in endothelial cells with a ROS probe after LPI treatment. The results indicated that LPI promoted ROS production. Furthermore, to investigate the association between ROS and endothelial cell activation, we measured ICAM1 levels after mito-TEMPO administration. We found that the upregulation of ICAM1 induced by LPI was significantly inhibited by mito-TEMPO, suggesting that mtROS may participate in endothelial cell activation. There are many reasons for the increase in ROS, such as the failure of mitochondrial antioxidant functions, mitophagy dysfunction and so on [21]. SOD2, as a member of superoxide dismutase, plays an important role in the antioxidant processes. Thus, we detected the level of SOD2. The results showed that the activity and protein level of SOD2 were significantly reduced. Above all, these data indicated that SOD2 is responsible for increasing mtROS levels in LPI-induced endothelial cell activation.

Autophagy is a double-edged sword. Moderate autophagy levels of autophagy are beneficial for cell survival. However, high levels of autophagy may also be involved in the development of AS. Autophagy plays an important role in endothelial cell integrity. Dysregulation of autophagy, such as in endothelial cells, has shown to be associated with diverse types of pathologic conditions. Studies have shown that endothelial cell-specific knockout of atg5 could result in capillary rarefaction and accelerated diabetic nephropathy [22]. In endothelial-specific atg5 knockout mice, the disruption of endothelial autophagy could lead to significant pathological IL6-dependent EndMT and organ fibrosis [23]. IL-37 reduced inflammation and apoptosis of atherosclerotic endothelial cells by enhancing autophagy [24]. The activation of endothelial autophagy transfers miR-204-5p from endothelial cells to smooth muscle cells via exosomes, which prevents endothelial apoptosis and alleviates smooth muscle cell calcification [25]. Deletion of Atg5 or Atg7 results in the impaired *in vitro* and *in vivo* stimulated secretion of vWF [26]. Here, we studied the role of LPI-induced autophagy in AS. Currently, most researchers have focused on the LPI-GPR55 signalling pathway. In our study, we studied downstream signalling molecules of LPI-GPR55. RNA-Seq analysis revealed that LINC01235 expression increased after LPI treatment. Furthermore, we demonstrated that LINC01235 suppressed autophagy. LINC01235 is a novel regulator of AS.

Numerous studies have shown that LINC01235 can be combined with miRNAs, such as miR-6852-5p [27]. Until now, research on LINC01235 has mainly focussed on cancer. LINC01235 can promote GC cell metastasis via EMT and function as a prognostic biomarker [28]. LINC01235 is related to the prognosis of breast cancer [29]. However, there have been no studies on LINC01235 in cardiovascular disease. We first clarified the role of LINC01235 in AS. And using various methods, we found that LINC01235 sponged miRNA-224-3p and miRNA-224-3p targeted RABEP1. RABEP1 is involved in endosome homeostasis [30]. There was no research on RABEP1 in vascular cells. In our study, knockdown RABEP1, the level of autophagy was elevated. Therefore, RABEP1 may serve as a novel autophagy regulatory factor in AS.

Endothelial cells, vascular smooth muscle cells and macrophages are the three main cell types that constitute atherosclerotic lesions. Studies have reported that many LncRNAs are involved in regulating these cellular functions. LncRNA-p21 enhanced the transcription activity of p53 by interacting with MDM2 and then induced VSMC apoptosis [31]. LncRNA-Mexis increased the expression of ABCA1 to promote macrophage cholesterol efflux, which inhibited AS [32]. Under ischaemia or reperfusion conditions, lncRNA APF regulated autophagy by sponging miR-188-3p [33]. CERNA1 inhibited VECs apoptosis by miR-4707-5p/API5 and miR-4767/BCL2L12 [34]. Due to the poor stability of lncRNAs, there are challenges in studying specific biological functions and mechanisms. Moreover, the conservatism of lncRNA is poor, and some lncRNA is only expressed in a certain species. Therefore, it is difficult to study the mechanism of lncRNA both *in vivo* and *in vitro*. LINC01235 is expressed in tumours and ECs [28,35]. There are no homologous genes in mice. Although lncRNAs are not highly conserved across different species, the presence of upstream or downstream super conserved elements in some lncRNAs may provide insights for studying the cross-species translation function of lncRNAs [36].

In brief, we concluded that LPI targeted GPR55 and promoted endothelial cell activation. In addition, LPI suppressed autophagy through increasing the expression of LINC01235. LINC01235 combined with miRNA-224-3p, which can reduce the expression of RABEP1. Above all, we identified a new factor-LINC01235 and clarified its mechanism in HAECs.

## Data Availability

The data that support the findings of this study are available from the corresponding author, XYH, upon reasonable request.
